# Targeted Drug Delivery for Sustainable Crop Protection: Transport and Stability of Polymeric Nanocarriers in Plants

**DOI:** 10.1002/advs.202100067

**Published:** 2021-03-19

**Authors:** Sebastian J. Beckers, Alexander H. J. Staal, Christine Rosenauer, Mangala Srinivas, Katharina Landfester, Frederik R. Wurm

**Affiliations:** ^1^ Max‐Planck‐Institut für Polymerforschung Ackermannweg 10 Mainz 55128 Germany; ^2^ Department of Tumor Immunology Radboud Institute for Molecular Life Sciences Radboud University Medical Center Geert Grooteplein 26/28 Nijmegen 6525GA The Netherlands; ^3^ Cenya Imaging BV Tweede Kostverlorlenkade 11h Amsterdam 1052RK The Netherlands; ^4^ Sustainable Polymer Chemistry Group MESA+ Institute for Nanotechnology Faculty of Science and Technology Universiteit Twente PO Box 217 Enschede 7500AE The Netherlands

**Keywords:** agrochemical, drug delivery, miniemulsion, nanocarriers, plant protection

## Abstract

Spraying of agrochemicals (pesticides, fertilizers) causes environmental pollution on a million‐ton scale. A sustainable alternative is target‐specific, on‐demand drug delivery by polymeric nanocarriers. Trunk injections of aqueous nanocarrier dispersions can overcome the biological size barriers of roots and leaves and allow distributing the nanocarriers through the plant. To date, the fate of polymeric nanocarriers inside a plant is widely unknown. Here, the in planta conditions in grapevine plants are simulated and the colloidal stability of a systematic series of nanocarriers composed of polystyrene (well‐defined model) and biodegradable lignin and polylactic‐*co*‐glycolic acid by a combination of different techniques is studied. Despite the adsorption of carbohydrates and other biomolecules onto the nanocarriers’ surface, they remain colloidally stable after incubation in biological fluids (wood sap), suggesting a potential transport via the xylem. The transport is tracked by fluorine‐ and ruthenium‐labeled nanocarriers inside of grapevines by ^19^F‐magnetic resonance imaging or induced coupled plasma – optical emission spectroscopy. Both methods show that the nanocarriers are transported inside of the plant and proved to be powerful tools to localize nanomaterials in plants. This study provides essential information to design nanocarriers for agrochemical delivery in plants to sustainable crop protection.

## Graphical abstract

Nanocarrier‐mediated release of agrochemicals in planta is a promising approach to treat plant diseases and to reduce spraying of chemicals. This paper investigates the transport of a diverse library of polymeric nanocarriers in vitro and in planta rendering drug delivery in plants with nanocarriers a promising tool for future agriculture.

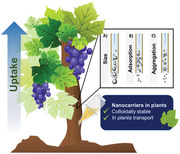

## 1 Introduction

Several million tons of agrochemicals are released into the environment every year.^[^ ^1^, ^2^ ^]^ Sustainable delivery systems for crop protection and minimal drug dosage are urgently needed and cannot be obtained with conventional spraying. Spraying only applies the drug onto the leaves, which leads to a high wash off effect and the transport into the plant is limited. One promising approach is the injection of agrochemical‐loaded nanocarriers for an “on‐demand” and target‐specific release of pesticides or fertilizers.^[^ ^3^ ^]^ Even if some in planta studies had been reported for trunk injections,^[^ ^4^, ^5^ ^]^ neither the colloidal stability of polymeric nano‐ or microcarriers inside of the plant nor the in planta transport after the injection are known. Previous studies focused on the transport and fate of inorganic nanoparticles or carbon materials but not biodegradable drug carriers.^[^ ^6^ ^]^ Thus, a detailed understanding of the behavior of polymeric nanocarriers in planta is essential, but still missing, to be able to design new agrochemical delivery systems for sustainable agriculture. This article investigates the colloidal stability and the transport of polymeric nanocarriers in young grapevines to set a basis for nanocarrier‐mediated drug delivery in plants.

To date, agrochemicals are mainly distributed by the spraying of drug formulations onto the plants. Despite advanced formulations that enhance the adhesion to leaf surfaces^[^ ^7^ ^]^ or the development of effective automated spraying devices,^[^ ^8^ ^]^ spraying is still a potential risk to the environment and the farmer. Agrochemicals can be washed off by rain and wind, and they might accumulate into the soil or reach the groundwater.^[^ ^2^ ^]^ Such contamination threatens insects, soil organisms and wild herbs, but might also contaminate crops and kettle or might be taken up by consumers.^[^ ^2^, ^9^ ^]^ In addition, several devastating trunk diseases with significant consumer impact cannot be treated by spraying from the outside, as the pest is located inside of the trunk.^[^ ^10^, ^11^ ^]^

A sustainable alternative to conventional crop protection might be the utilization of nanotechnology, in analogy to human medicine.^[^ ^12^ ^]^ Until now, in particular, inorganic metal nanoparticles (e.g., from gold, silver, zinc‐, titanium‐, or copper oxide) have been studied due to their intrinsic toxicity to plant pathogens.^[^ ^13^ ^]^ For example, aggregation of CdSe/ZnS nanoparticles stabilized with organic molecules was detected in ryegrass.^[^ ^14^ ^]^ In contrast, carbon nanotubes permeated into the roots of intact living mustard plants and were then transported throughout the plants.^[^ ^15^ ^]^ Besides these examples, some more advanced drug delivery systems had been developed based on biopolymers such as chitosan,^[^ ^16^, ^17^ ^]^ lignin,^[^ ^5^, ^18^, ^19^, ^20^ ^]^ xylan,^[^ ^21^ ^]^ and alginate,^[^ ^22^ ^]^ or from synthetic polymers such as poly(allyl amine) hydrochloride^[^ ^23^ ^]^ or poly(*ε*‐caprolactone).^[^ ^16^ ^]^

For optimal performance of a drug delivery vehicle in planta, transport through a series of physiological and chemical barriers within the plant needs to be understood. Fundamental studies on transport of nutrients and contaminants via the phloem can be used as a basis.^[^ ^24^ ^]^ In particular, size is considered one of the major restrictions for penetration into plant tissues.^[^ ^25^, ^26^, ^27^, ^28^ ^]^ Studies underlined that only relatively small nanomaterials are taken up by most roots (smaller than 50 nm) or by penetration of leaf surfaces (smaller than 10 nm).^[^ ^29^, ^30^ ^]^ Hence, polymer‐based nano‐ and microcarriers, which typically have diameters above 100 nm, cannot be taken up by the plants when sprayed. This small size requirement can be a limiting factor in the development of suitable carriers.

**Scheme 1:**
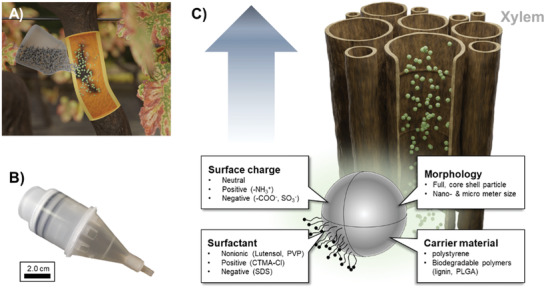
A) Schematic illustration of a trunk injection of a nanocarrier dispersion. B) Commercially available injector applicable for trunk injections supplied from Tree Tech Microinjection Systems (FL, US). C) Nanocarriers after injection into the xylem of plants and possible factors that influence nanocarrier stability and transport.

One promising alternative to overcome the size limitation is a trunk injection directly into the vascular tissue (**Scheme** **1**A,B). Trunk injection can be achieved by drilling a hole into the plant and subsequent use of injector devices or syringes;^[^ ^4^ ^]^ more recent developments use microneedles, which reduce the size of the injection point.^[^ ^31^ ^]^ When injected into the trunk, nanocarriers should be transported via the xylem or phloem, which have diameters of several micrometers.^[^ ^5^ ^]^ Trunk injections can broaden significantly the scope for nano‐ and microcarrier‐mediated drug delivery inside of plants.^[^ ^25^, ^32^ ^]^ However, colloidal stability in the wood sap is crucial for the successful transport inside of the plant, otherwise, aggregation occurs after injection, which might be used for the formation of a drug depot. The colloidal stability inside of the plant is determined by the chemical design, surfactant, or surface charges that interact with the complex in planta conditions.

Herein, we report the behavior of polymeric nanocarriers in simulated wood saps and in in planta conditions by a unique set of experiments. First, we simulated the in planta conditions that a nanocarrier would encounter after trunk injection using wood extracts of commercially relevant fruiting plants (grapevine, apple, and peach). A library of model‐polystyrene nanocarriers (different surfactants, surface charges, and sizes, see Scheme 1C) was investigated regarding their colloidal stability and surface modification after incubation in the respective biological fluids by using dynamic light scattering. Additionally, biodegradable lignin and polylactic‐*co*‐glycolic acid (PLGA) nanocarriers have been considered as versatile drug delivery vehicles,^[^ ^33^ ^]^ we also tested these systems herein.^[^ ^5^, ^18^, ^34^, ^35^, ^36^ ^]^ Despite the formation of a bio‐corona, all investigated nanocarriers remained macroscopically stable, however, dynamic light scattering proved some aggregation for cationic nanocarriers. We applied biomedical imaging techniques to study carrier biodistribution and pharmacokinetics, using nanocarriers suitable for both imaging and drug delivery. All nanocarriers were translocated in in planta studies, which was followed by tracing labeled nanocarriers either by induced coupled plasma – optical emission spectroscopy (ICP‐OES) (ruthenium‐labeled) or by ^19^F‐magnetic resonance imaging (MRI) (fluorine‐labeled).^[^ ^37^ ^]^ The data prove the potential of polymeric nanocarriers as delivery vehicles for future agriculture.

## 2 Results and Discussion

The colloidal stability of polymeric nanocarriers for agrochemical release inside of the plant determines the transport properties through the vascular tissue of the plant. In general, two different scenarios are conceivable and have been studied in this paper.

### 2.1 Depot

After injection into the trunk of the plant, the dispersion aggregates, which would probably lead to the formation of a “drug depot”. A sustained release of the drug by diffusion could occur. However, for a pathogen‐induced drug release, the fungi or bacteria would need to penetrate the vascular system to reach the nanocarrier depot to initiate the drug release. Alternatively, the dispersion would need to be injected directly into the infected tissue.

### 2.2 Transport

After injection into the trunk, the nanocarrier dispersion remains colloidally stable and can be transported through the plant reaching the place of infection. However, if the colloids move to the shoots or the leaves, they might be lost by pruning procedures or by shedding the leaves after summer, which might reduce the long‐term protection.

For efficient drug delivery over several years, probably a combination of both scenarios is desirable. Lignin nanocarriers loaded with fungicides had been previously studied in planta for the treatment of the grapevine trunk disease Esca.^[^ ^5^ ^]^ In our recent work, we injected aqueous nanocarrier dispersions into grapevine plants, in which lignase‐producing fungi led to an “on‐demand” release of encapsulated pesticides.^[^ ^5^, ^18^, ^34^ ^]^ However, the colloidal stability or transport inside of the plants of these lignin nanocarriers had not been investigated. We believe that this concept is a general approach for agrochemical release in planta.

## 3 Simulation of In Planta Conditions by Wood Saps

### 3.1 Characterization of Wood Saps

**Figure 1:**
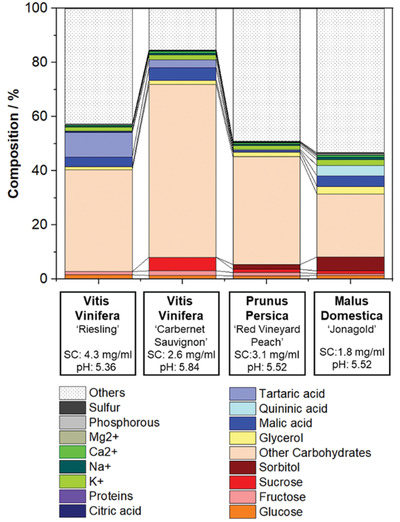
Composition of wood saps from four different commercially relevant fruiting plants (determined by HPLC, ICP‐OES, GC‐MS, Anthrone, and Pierce assay).

To understand the in planta conditions after nanocarrier injection, four different wood saps of relevant fruiting plants were used to simulate the in planta situation. Taking the global crop sizes of fruits into account, we chose to analyze the saps of *Malus domestica* (apple, 89 Mio tons, rank 3 in 2016), *Vitis vinifera* (grapes, 77 Mio tons, rank 4), and *Prunus persica* (peach, 25 Mio tons, rank 10). The wood saps were produced by aqueous extraction of lyophilized wood chips and are mixtures of xylem and phloem saps. Wood extraction is a well‐established method to isolate plant‐based solutes, e.g., for phytomedical applications.^[^ ^13^, ^38^, ^39^ ^]^ According to the literature, wood sap is mainly composed of carbohydrates, organic acids, salts, and trace amounts of proteins as well as amino acids.^[^ ^6^, ^40^, ^41^ ^]^ The wood saps were investigated regarding their chemical composition with high‐pressure liquid chromatography (HPLC), gas chromatography‐mass spectrometry (GC‐MS), inductively coupled plasma optical emission spectrometry (ICP‐OES), and Anthrone and Pierce assays (**Figure** **1** and Figure S1: Supporting Information). The quantification of carbohydrates by the Anthrone assay proved that 30–70% carbohydrates are one of the major species in the sap. To a high extent, these originate from the phloem, in which carbohydrates are produced during photosynthesis and translocated throughout the plant. By HPLC, glucose, fructose, and sucrose were identified in all wood saps, whereas sorbitol was found only in the extracts of peach and apple. In accordance with the literature, malate was found in remarkable amounts of up to 4.6% in all extracts. Further, tartaric acid was identified in extracts from “Riesling” wood by GC‐MS. Organic acids resulted not only in acid pH values of ≈5.5 but also affect the stability of colloids due to their ability to shield surface or surfactant charges. In contrast to nanocarrier‐mediated drug delivery in blood for biomedical application, the risk of protein adsorption and the resulting formation of aggregates is negligible in wood saps, as the protein amounts were below the detection limit of the Pierce assay (<10 µg mL^−1^). Further, potassium, sodium, magnesium, and calcium were quantified by ICP‐OES. Considering the literature, we assume the respective counter ions like nitrate, chloride phosphate and sulfate are contained in the wood sap additionally.^[^ ^40^ ^]^ The concentration was estimated by measuring the amount of phosphorus and sulfur by ICP‐OES respectively.

### 3.2 Nanocarrier Library

**Figure 2:**
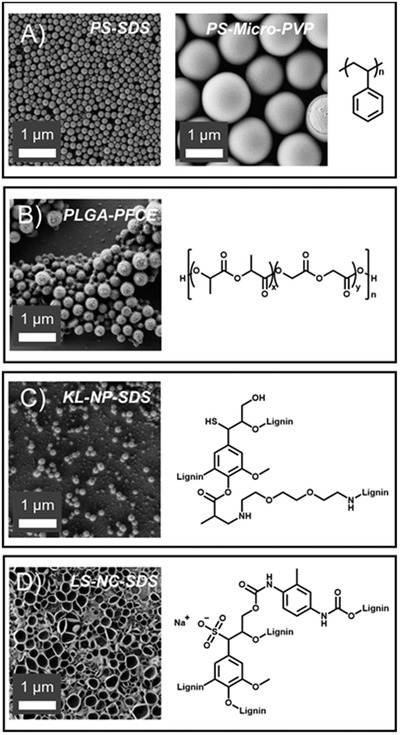
Scanning electron microscopy images showing the morphologies of A) polystyrene, B) PLGA, C) Kraft Lignin (KL), and D) lignin sulfonate (LS) nanocarriers; right shows a representative chemical structure of the nanocarriers' composition.

We prepared a systematic library of nanocarriers with different surface chemistries and functionalities and studied their colloidal stability in the wood saps. Nanocarriers with different surface functionality were obtained by using different surfactants and introducing comonomers in the case of polystyrene (PS) nanoparticles. Polystyrene nanoparticles are a well‐established model system to study interactions of nanocarriers with blood serum or plasma,^[^ ^42^ ^]^ while to the best of our knowledge, nothing had been reported about the colloidal stability of nanocarriers in wood saps. The model PS nanocarriers were stabilized either by the anionic surfactant SDS, the cationic CTMA‐Cl, or by the nonionic surfactant Lutensol AT50. Additionally, we prepared copolymers of styrene with acrylic acid and 2‐aminoeethyl methacrylate hydrochloride, which led to additional covalently‐bond anionic (in the case of acrylic acid) or cationic charges (in the case of ‐aminoeethyl methacrylate hydrochloride) on the nanocarriers. The characterization data of the PS‐NP library are listed in **Table** **1** and **Figure** **2**. Additionally, we prepared biodegradable nanocarriers as closer models for in planta drug delivery, based on lignin and poly(lactide‐*co*‐glycolide) (PLGA). As both materials are bio‐based and biodegradable, they are promising candidates for drug delivery and enzyme‐triggered release in plant protection.^[^ ^5^, ^34^, ^43^, ^44^ ^]^

**Table 1:** Characterization of the nanocarrier library prepared for this study: polystyrene (PS), Kraft lignin (KL), lignin sulfonate (LS), and poly(lactide‐*co*‐glycolide) (PLGA) nanocarriers and their particle size, polydispersity (PDI), *ζ*‐potential, and surface charge density

Nanocarrier	*R*_h_ [nm]^a)^	PDI^a)^	*ζ*‐pot [mV]^b)^	Groups [nm^2^]^c)^
PS‐SDS	90	0.002	−44 ± 1	–
PS‐Lut.	110	0.005	−10 ± 2	–
PS‐CTMA	120	0.048	+23 ± 1	–
PS‐NH_2_‐Lut.	100	0.167	+7 ± 1	0.1
PS‐COOH‐Lut.	80	0.144	−34 ± 3	0.6
PS‐Micro‐PVP	750^d)^	–	−6 ± 2	–
KL‐NP‐SDS	90	0.256	−36 ± 3	–
LS‐NC‐SDS	150	0.416	−32 ± 1	–
KL‐NP‐Lut.	100	0.270	−20 ± 1	0.9
LS‐NC‐Lut.	160	0.407	−28 ± 1	–
PLGA‐PFCE	110	0.381	−14 ± 1	–

^a)^ Measured by dynamic light scattering

^b)^ Measured by a Zetasizer

^c)^ Measured by a particle charge detector

^d)^ Measured by SEM.

Herein, we studied two lignin nanocarriers, which differ in their chemical functionality and their surface charge. Nanocarriers from a direct miniemulsion using methacrylated Kraft lignin (KL) for the encapsulation of hydrophobic cargo were prepared. These nanocarriers had 0.9 anionic charges per nm^2^ of particle surface and showed a negative *ζ*‐potential of −20 mV, due to the negatively charged phenolate and carboxylate groups in lignin's structure (Table 1). As another example, lignin‐nanocarriers were prepared by an inverse miniemulsion, which allows the encapsulation of hydrophilic cargo. The crosslinking of a lignin sulfonate sodium salt (LS) with toluene diisocyanate at the interface resulted in nanocarriers with a core–shell structure and a *ζ*‐potential of −28 mV because of phenolic units and sulfonic acid groups of the lignin sulfonate.

PLGA (lactide and glycolide in an equimolar ratio) nanocarriers were prepared by the method of Srinivas et al.^[^ ^37^ ^]^ The nanocarriers were composed of a dense polymer matrix, in which the MRI contrast agent perfluoro‐15‐crown‐5‐ether (PFCE) was embedded, of about 200 nm diameter with a zeta potential of −14 mV. PLGA nanocarriers are heavily researched for biomedical applications as the (enzymatic) hydrolysis of its ester linkages not only allows for a controlled release of encapsulated cargo, but also leads to the biocompatible degradation products lactic and glycolic acid.^[^ ^35^ ^]^ Both compounds occur naturally in plants. Likewise, in vitro tests proved that PLGA nanocarriers are non‐phytotoxic for plant cells (but were not tested herein).^[^ ^45^ ^]^

### 3.3 Wood‐Sap Nanocarrier Interactions

**Figure 3:**
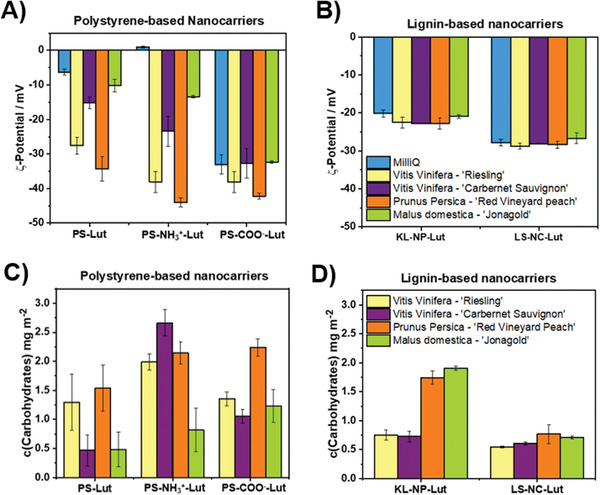
A,B) Nanocarriers incubated in wood saps: *ζ*‐potentials and C,D) carbohydrate adsorption (after 1 h incubation at RT). All nanocarriers were stabilized with the nonionic surfactant Lutensol AT50.

The interactions of the different nanocarriers with the wood extracts of *Prunus persica* (peach) cv. “Red vineyard peach”, *Malus domestica* (apple) cv. “Jonagold”, *Vitis vinifera* (grapevine, red grapes) cv. “Carbernet Sauvignon” and *Vitis vinifera* (grapevine, white grapes) cv. “Riesling” were studied. The nanocarriers were incubated in the wood sabs to mimic the in planta conditions after the injection into a plant and the *ζ*‐potentials were measured, which indicated the adsorption of charged compounds on the nanocarriers’ surface. In general, the *ζ*‐potentials decreased for non‐ionic (PS‐Lut) and positively charged nanocarriers (PS‐NH_3_^+^‐Lut) to values between −10 and −40 mV, indicating the adsorption of organic acids and other anions (e.g., phosphate, nitrate, and sulfate or interfacially active compounds), whereas the *ζ*‐potential remained relatively constant in the case of all nanocarriers with an originally negative *ζ*‐potential (PS‐COO^−^Lut (≈−30 mV), KL‐NP‐Lut (≈−20 mV), and LS‐NC‐Lut (≈−30 mV), **Figure** **3**A,B). The amount of surface‐adsorbed carbohydrates was quantified by the Anthrone assay and found to be ≈0.5–3.0 mg m^−2^, which means a surface coverage of 1–8% after the applied procedure (considering an area of 0.78 nm^2^ for a single glucose molecule (Figure 3C,D).

### 3.4 Colloidal Stability of Nanocarriers in Wood Sap

Dynamic light scattering was used to monitor the colloidal stability of the nanocarrier dispersions after incubation in the wood saps. For nanocarriers in wood saps from grapevines and apple, a bi‐exponential fit for the evaluation was used, as the wood saps did not contain additional scattering (Equation (1)). In contrast, extracts from peach wood exhibited strong scattering also without the addition of nanocarriers (max 0.8% of scattering; removal by centrifugation or filtration impossible). To evaluate the colloidal stability of the nanocarriers in the peach extract, we used the protocol of Rausch et al. for data evaluation, which was previously used to analyze the colloidal stability of nanocarriers in blood serum.^[^ ^46^ ^]^ Herein, we expanded this method to wood saps for the first time, proving the technique's versatile fields of application. Briefly, the autocorrelation function of the biological fluid (Equation (2)) is described as a sum of three exponentials where *a*_i_ is the amplitude and *τ*_i_ = 1/(*q* ^2^ *D*_i_) is the decay time containing the scattering vector *q* and the Brownian diffusion coefficient *D*_i_.

(1) $$g_{1,NP}=a_{1,NP}\cdot e^{-\frac{t}{\tau_{1,NP}}}+a_{2,NP}\cdot e^{-\frac{t}{\tau_{2,NP}}}$$

(2) $$g_{1,WS}=a_{1,WS}\cdot e^{-\frac{t}{\tau_{1,WS}}}+a_{2,WS}\cdot e^{-\frac{t}{\tau_{2,WS}}}+a_{3,\;WS}\cdot e^{-\frac{t}{\tau_{3,WS}}}$$

After determining both scattering profiles separately, a mixture of nanocarriers and wood sap was analyzed. If the nanocarriers stay colloidal stable, the corresponding autocorrelation function can be fitted as a sum of *g*_1,NP_ and *g*_1,WS_ (Equation (3)). However, a sufficient fitting is impossible in the case of aggregation and an additional exponential term, describing the scattering of the new species must be added (Equation (4)).

(3) $$g_{1,\mathrm{Mix}}=f_{\mathrm{NP}}g_{1,\mathrm{NP}}+f_{\mathrm{WS}}g_{1,\mathrm{WS}}$$

(4) $$g_{1,\mathrm{Mix}}=f_{\mathrm{NP}}g_{1,\mathrm{NP}}+f_{\mathrm{WS}}g_{1,\mathrm{WS}}+f_{\mathrm{Agg}}g_{1,\mathrm{Agg}}$$

**Figure 4:**
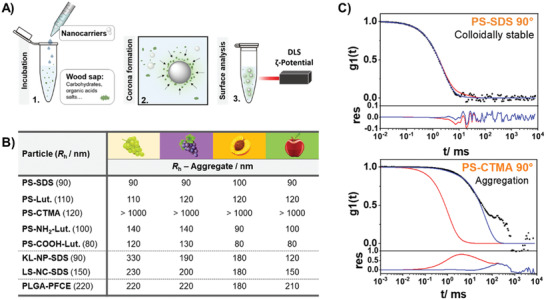
Colloidal stability of nanocarriers in wood saps. A) Procedure to investigate interactions of wood saps and nanocarriers. B) Colloidal stability of nanocarriers after 24 h incubation in different wood saps mimicking the in planta conditions, measured by dynamic light scattering (DLS). C) Two examples showing the autocorrelation function of colloidally stable nanocarriers (PS‐SDS) and the formation of aggregates after incubation in “Riesling” sap (PS‐CTMA).

Almost all nanocarrier dispersions remained stable after incubation in the wood extract. Despite the varying compositions for grapevine, apple, and peach, only marginal changes regarding the size distribution of each dispersion were observed (**Figure** **4**). We, therefore, assume that these nanocarriers also remain colloidally stable inside of a plant and can be distributed through the vascular plant tissue. The only exception was CTMA‐Cl stabilized PS‐particles, which agglomerated and formed macroscopic aggregates due to shielding of CTMA's positive charges by anionic solutes from the wood sap. If the aggregates get larger than the micrometer‐sized channels of the vascular tissue the transport might be hindered and a depot could be formed.

## 4 In Planta Biodistribution

The transpiration stream generated by the evaporation of water over the leaf surface enables the plant to transport water and nutrients through the vascular tissue in the trunk. To understand if this mechanism can be used for the distribution of nanocarriers in planta, we monitored the transport of labeled nanocarriers either by inductively coupled plasma emission spectroscopy or by ^19^F magnetic resonance imaging.

### 4.1 Elemental Analysis by ICP‐OES

**Figure 5:**
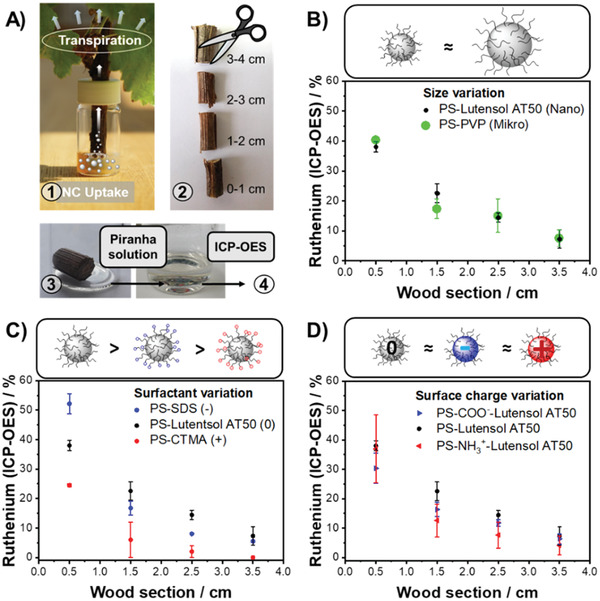
Uptake and transport of ruthenium‐labeled nanocarriers through Riesling cuttings: A) Experimental setup to follow the transport: 1. Uptake and distribution for 7 days. 2. Cutting into four segments. 3. Dissolution in “Piranha solution”. 4. Quantification of ruthenium. Distribution profile of ruthenium‐labeled polystyrene nanocarriers monitored by ICP‐OES depending on B) size, C) surfactant, D) surface

The bio‐distribution of a systematic library of ruthenium‐loaded PS model nanocarriers and biodegradable lignin nanocarriers in grapevine cuttings (*Vitis vinifera* cv. “Riesling”; length: 4 cm, diameter: 0.8 cm) was studied by elemental analysis. PS model nanocarriers allowed a systematic variation of surfactant, surface charge, and diameter, while lignin nanocarriers represent a promising formulation for sustainable plant protection. Before the miniemulsification, ruthenocene was added to the styrene. During the miniemulsion polymerization, the ruthenocene was loaded into the nanocarriers, which acted as an ICP‐probe for the biodistribution. The plants were immersed into a nanocarrier dispersion and after seven days, the stem of each plant was cut into 1 cm long pieces (**Figure** **5**A). Each piece of wood was dissolved in a mixture of hydrogen peroxide and concentrated sulfuric acid (“piranha solution”) and the ruthenium amount was analyzed by ICP‐OES (Table S1, Supporting Information).

The amount of the ruthenium correlated with the trunk height of each segment and yielded a nanocarrier‐specific ruthenium transport profile: In all cases, the ruthenium content was high for segments located at lower parts of the trunk and decreased up to the leaves, where no or only trace amounts were detected. However, in almost all wooden segments ruthenium was detected, indicating a transport through the trunk. Surfactant, surface charge, or size influenced the transport kinetics only slightly. Figure 5B shows the transport profiles for PS nanocarriers, stabilized with nonionic, anionic, and cationic surfactants: CTMA‐Cl stabilized nanocarriers exhibited the lowest transport of this series. This correlates with the formation of aggregates after the incubation in wood sap observed by dynamic light scattering (DLS). Nanocarriers stabilized with the anionic SDS indicated slightly higher amounts of ruthenium in higher parts of the cutting, followed by the nonionic nanocarriers that exhibited the highest values for ruthenium. Very similar transport profiles were observed when nanocarriers without, or with positive or negative surface charges were used (all stabilized by nonionic surfactant), indicating a slightly lower transport of ruthenium for ionically charged nanocarriers, compared to the nonionic analogs (Figure 5D). Likewise, the anionic lignin nanocarriers did not significantly differ from PS‐based dispersion regarding their ruthenium distribution profile (Figure S2, Supporting Information), suggesting that various nanocarriers are applicable for drug delivery in plants. Moreover, we compared the bio‐distribution of polystyrene nanocarriers (Ø 200 nm) prepared by the miniemulsion approach with polystyrene microcarriers (Ø 1500 nm) synthesized by a simple dispersion polymerization (Figure 5B). Although differing in size, for both carrier‐types an almost identical ruthenium profile was detected, which correlated with microscopy images showing diameters of ≈20 µm for the xylem vessels of the herein used test plants. We assume that controlled drug delivery inside of plants is possible when the drug delivery vehicle is significantly smaller than the diameter of the plant's transport vessels and aggregation is avoided.

### 4.2 Imaging by ^19^F MRI

**Figure 6:**
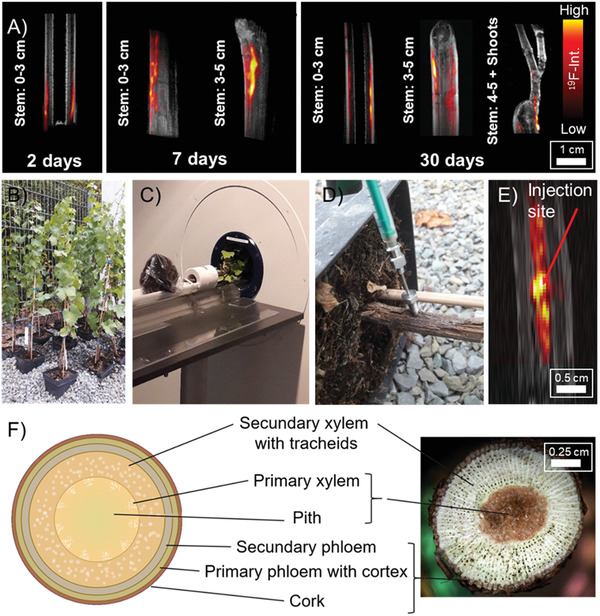
A) ^19^F‐^1^H overlay MRI images of grapevine cuttings 2, 7, and 30 days after uptake of perfluoro‐15‐crown‐5‐ether (PFCE) loaded PLGA nanocarriers via the transpiration stream. For uptake, the cuttings were immersed in nanocarriers dispersion as shown in Figure 5A. PFCE allows the localization of the nanocarriers within the plant. The concentration of fluorine (false color) is shown in yellow (high), red (low), black/gray (anatomy or ^1^H image). B) Potted “Riesling” test plants. C) Grapevine test plant in MRI. D) Injection of a concentrated nanocarrier dispersion into the trunk of a potted grapevine test plant. E) ^19^F‐^1^H overlay MRI image showing the injection profile after an injection of 0.2 µL of a 10 wt% PFCE‐PLGA dispersion via syringe. No further transport was observed 7 days after the treatment. F) Schematic illustration and photo of a cross‐section of a grapevine shoot used to study the nanocarrier uptake.

To visualize the transport of the nanocarriers through the vascular tissue of grapevine cuttings, we utilized perfluoro‐15‐crown‐5‐ether (PFCE) loaded PLGA nanocarriers, which were traced by noninvasive ^19^F MRI. The fluorine‐containing cargo acts as a contrast agent and allows a specific and quantitative localization by ^19^F MRI, as plant tissue is almost free of fluorine.^[^ ^47^ ^]^ According to scanning electron microscopy (SEM) and DLS, the morphology of the nanocarriers does not change in acidic “Riesling” wood sap (pH of 5.4), which proves the stability of the PLGA‐matrix against hydrolysis under these conditions. Grapevine shoots were immersed into dispersions of PFCE‐loaded PLGA nanocarriers (*Vitis vinifera* cv. “Riesling”; length: 4 cm, diameter: 0.8 cm; as shown in Figure 5A) or a nanocarrier dispersion was injected with a syringe into the trunk of potted grapevine plants (*Vitis vinifera* cv. “Riesling”; length: 25–30 cm, diameter: 0.8–1.0 cm; **Figure** **6**B). The transport of the nanocarriers was visualized after 2, 7, and 30 days using MRI on a preclinical system.

When immersing the Riesling cuttings into a PFCE‐loaded nanocarrier dispersion, a high ^19^F resonance signal was detected especially in tissue with strong sapflow (trunk side from which the leaf was grown). This signal was located mainly between bark and pith, which suggests that xylem transport was used as a primary distribution mechanism. After two days, a ^19^F MRI signal was detected at a trunk height of 1.5 cm proving the transport of the nanocarriers inside the trunk (Figure 6A, left image). 5 days later, a strong ^19^F MRI resonance signal was detectable in the complete shoot (Figure 6A, middle image), which extended after 30 days also to the fine stems of the leaves, proving a fast distribution of the nanocarriers inside of the cutting (Figure 6A, right image). In correlation with the results obtained from ICP‐OES analysis, no fluorine signal was detected in the leaves. We assume that the nanocarriers remain in wooden trunk tissue.

When the nanocarrier dispersion was injected into a trunk of potted Riesling plants, an intense ^19^F MRI signal was detected in the pith (Figure 6D). The signal reached ≈4 cm above and 6 cm below the point of injection showing that the nanocarriers were pressed through plant tissue. After a week, the fluorine distribution profile showed a slight increase of fluorine at higher parts of the plant, but the major signal remained at the point of the injection, which most likely is due to the weaker sapflow in the pith (Figure 6E and Figure S3: Supporting Information). These results show the importance of the method of application for nanocarrier based plant therapy, which needs further studies also to understand the influence on plant physiology after injection. Transport of the nanocarrier via the xylem results in relatively rapid dispersion of the nanocarrier throughout the plant. Whereas a direct bulk injection into the pith might result in a local depot that is transported much slower. Moreover, the nanocarrier did not show migration from the pith to the xylem or vice versa during the monitored timeframe. These findings highlight the importance of imaging in the development of targeted drug delivery. To our knowledge, this is the first application of an imaging nanoparticle to assess the transport of therapeutic nanocarriers through plants. Application of 19F MRI in plants has up till now been limited to hydrophilic fluorinated molecules as model drugs such as trifluoroacetate.^[^ ^48^ ^]^

## 5 Conclusion

This work presents the first investigation of the stability and the transport of polymeric nanocarriers in plants: wood saps proved to be suitable to simulate real in planta conditions. Most investigated nanocarriers did not aggregate significantly when incubated in the extracts of grapevine, apple, or peach and might be suitable as drug delivery vehicles in plants. To visualize the transport in living plants, we used Riesling cuttings or young potted Riesling plants. We developed two techniques based on elemental mapping (by ICP‐OES) or imaging (by ^19^F MRI). Chemical composition, size, surface charge, or surfactant of the nanocarriers were varied systematically and in all cases, transport through the vascular tissue of our test plants was detected. The surface charges seemed to play a minor role in transport efficiency when Riesling cuttings were incubated in nanocarrier dispersions. Injection in potted Riesling plants was slower, compared to incubation of cuttings directly in nanocarrier dispersions but effective transport of nanocarriers was visualized in both cases. Non‐invasive imaging is important to understand the in planta fate of the drug, which might further be combined with the mild recently developed microneedle‐techniques for injection of drugs into living plants.^[^ ^31^ ^]^ To conclude, polymeric nano‐ and microcarriers are a versatile strategy to develop targeted drug delivery inside of plants and will allow the development of sustainable delivery of agrochemicals in the future.

## Experimental Section

##### Materials

The following materials were used: Anthrone, hexadecane, 2,2'‐azobis(2‐methylbutyronitrile) (V59), sodium dodecyl sulfate (SDS), cetyltrimethylammonium chloride solution (25 wt% in water, CTMA‐Cl), acrylic acid, lignin sulfonic acid sodium salt (product number: 471 038), Kraft lignin (product number: 370 959), polyvinylpyrrolidone (PVP, 24 kg mol^−1^), toluene diisocyanate (TDI) and 2,2’‐(ethylenedioxy)bis(ethylamine) were obtained from Sigma Aldrich. 2‐Aminoethyl methacrylate hydrochloride and ruthenocene were products of Acros Organics and STEM chemicals, respectively. Lutensol AT50 was supplied from BASF SE. All chemicals were used without further purification. Polystyrene was obtained from Sigma Aldrich and was distilled before use. PFCE‐loaded PLGA nanocarriers were prepared according to the protocol of Srinivas et al. and were supplied by the Radboudumc (NL).

*Vitis vinifera* cv. “Riesling” cuttings and plants were supplied by the DLR (Neustadt a. d. Weinstraße) and Antes Weinbau‐Service GmbH (Heppenheim). Grafting wax purchased from W. Neudorff GmbH KG (Emmerthal, Germany) was used to seal the boreholes in the trunk. To mimic the in planta conditions after trunk injection, in February 2019, woodcut from the trunk of the following plants was collected: *Prunus persica* (Peach) cv. “Red vineyard peach”, *Malus domestica* (Apple) cv. “Jonagold”, *Vitis vinifera* (Grapevine) cv. “Carbernet Sauvignon” and *Vitis vinifera* (Grapevine) cv. “Riesling”.

##### Methods—Preparation of Wood Extracts

20 g of lyophilized wood chips (length: 1–3 cm; ≈66 g wet mass) were blended for 1 min with 100 mL of MilliQ water. Afterward, the mixture was filtered through a paper filter to separate the solid. Before use, the extract was filtered through a 0.45 µm syringe filter. The solute concentration was assumed ≈50% relative to in planta conditions.

##### Methods—High‐Performance Anion‐Exchange Chromatography with Pulsed Amperometric Detection (HPAEC‐PAD)

Wood extracts were analyzed using high‐performance anion‐exchange chromatography. The solutes were separated as anions under high alkaline conditions (pH > 12), coupled with pulsed amperometric detection. HPAEC‐PAD analysis was performed on a Shimadzu LC system equipped with two LC‐10Ai pumps, a DGU‐20A degassing unit, a SIL‐10Ai autosampler, a CBM‐20A controller and a CTO‐20AC column oven. An analytical anion‐exchange column of CarboPac MA1 (4 × 250 mm) in combination with a guard column of CarboPac MA1 (4 × 50 mm) at 20 °C was used. A Dionex ED40 Electrochemical detector was used for the detection of carbohydrates and sugar alcohols in pulsed amperometric mode through standard quadruple waveform (*t* = 0–0.40 s, *p* = 1.00 V; *t* = 0.41–0.42 s, *p* = −2.00 V; *t* = 0.43 s, *p* = 6.00 V; *t* = 0.44–0.50 s, *p* = −1.00 V). Eluent was prepared as the mobile phase, which consisted of 480 × 10^−3^ m NaOH solution. Isocratic elution was performed at 0.4 mL min^−1^. All the samples were filtrated through a 0,2 µm filter. Injection volume was 10 µL.

##### Methods—Gas Chromatography with Mass Spectrometry Detector (GC‐MS)

The dry samples were derivatized with 10 µL of ethoxyamine hydrochloride solution in pyridine and 20 µL of pyridine for 90 min at 40 °C. Subsequently, the samples were silylated for 50 min at 40 °C with 70 µL MSTFA. The samples were analyzed with a Shimadzu GCMS‐QP 2010 gas chromatograph coupled with a quadrupole mass analyzer. 1 µL aliquots of the samples were injected into a DB5‐MS capillary column (30 m x 250 µm I.D., 0.25 µm film thickness, Phenomenex, Germany) in split mode (1:10) using an AOC20i autosampler. The temperature of the injection system was 250 °C. The initial GC oven temperature was 70 °C, 5 min after injection the GC oven temperature was increased with 5 °C min^−1^ to 320 °C and held for 5 min at 320 °C. Helium was used as a carrier gas with a flow rate of 1 mL min^−1^. Detection was achieved using MS detection in electron impact mode and full scan monitoring mode (*m*/*z* 15–800). The temperature of the ion source was set at 200 °C and the transfer line at 275 °C. Identification was carried out by comparing the mass spectra with the NIST spectral library.

##### Methods—Bio‐Corona Formation in Wood Extracts

A dispersion (typically 1 wt%) with a calculated surface area of 0.05 m^2^ was added to 0.5 mL of wood sap and incubated at 25 °C for 1 h. Then, the dispersion was centrifuged (PS nanocarriers: 20k rpm, 30 min; Lignin nanocarriers: 10k rpm, 15 min) and the supernatant was replaced with MilliQ water. After resuspension of the pellet, the nanocarriers were washed by three further centrifugation steps and analyzed regarding surface modifications by *ζ*‐potential measurements and anthrone‐ plus malate assays.

##### Methods—Anthrone 620 nm Carbohydrate Quantification Assay

5 mg anthrone were dissolved in 1 mL of concentrated sulfuric acid. To 150 µL of the anthrone stock solution, 75 µL of a carbohydrate mixture was added. After incubation at 80 °C for 10 min, the absorbance was measured at 620 nm with a Tecan Infinite M1000 plate reader. The evaluation was performed relative to a d‐glucose calibration.

##### Methods—Pierce 660 nm Protein Quantification Assay

The protein concentration of wood saps was determined using a Pierce 660 nm protein assay (Thermo Fisher, Germany) following the instructions from the manufacturer. The absorbance was measured at 660 nm with a Tecan infinite M1000 plate reader.

##### Methods—Malate and Glycerol Quantification Assay

Malate was quantified enzymatically using Kit Nr. 10 139 068 035 from R‐biopharm. Kit Nr. 10 148 270 035 from R‐biopharm was used to quantify glycerol.

##### Methods—Dynamic Light Scattering

All DLS experiments were performed on an ALV/CGS3 spectrometer (ALV GmbH, Germany) consisting of an electronically controlled goniometer and an ALV‐5000 multiple tau full‐digital correlator. As a light source, a helium‐neon laser with a wavelength of 632.8 nm and output power of 25 mW (JDS Uniphase, USA, Type 1145P) was used. The measurement was performed in cylindrical quartz cuvettes (Inner diameter: 18 mm, Hellma, Germany) at room temperature for 5 times 30 s at 30°, 60°, 90°, 120°, and 135°. Analysis of the autocorrelation function was using either using a CONTIN or a HDRC algorithm.

To determine the colloidal stability of nanocarrier dispersions after injection into a plant stem, the wood extract was filtered through a 0.45 µm Millex‐LCR syringe filter. 50 µL was of a 1 wt% dispersion was then added to 150 µL of wood extract and incubated overnight at room temperature. Due to the negligible scattering contribution of wood saps from grapevine and apple, the data was evaluated using the CONTIN method, whereas extracts of peach wood, containing non‐removable aggregates, needed evaluation with the HDRC algorithm.^[^ ^46^ ^]^

##### Methods—*ζ*‐Potential Measurements

To determine the *ζ*‐potential, 20 µL of a 1 wt% dispersion was added to 2 mL of 1 × 10^−3^ m KCl solution. 1 mL of the mixture was filled in a cuvette and analyzed using a Zetasizer NanoZ (Malvern).

##### Methods—Scanning Electron Microscopy

SEM was performed on a Gemini 1530 (Carl Zeiss AG, Oberkochem, Germany) scanning electron microscope operating at 0.35 kV. The samples were prepared by casting diluted nanocarrier dispersions on silicon wafers.

##### Methods—Particle Charge Detection (PCD)

The number of positive and negative surface charges was determined by titration of a 0.1 mg mL^−1^ nanocarrier dispersion on a particle charge detector PCD 02 (Mütek Gmbh, Germany) combined with a Titrino Automatic Titrator 702 SM (Metronohm AG, Switzerland). Positive or negative charges were titrated either against 0.001 N solutions of the anionic poly(ethylene sulfonate) or cationic poly(diallyl dimethylammonium chloride) respectively.

##### Methods—In Planta Studies – Nanocarrier Uptake

Two experimental setups to investigate the transport of nanocarriers inside of a plant were used:

- Nanocarrier uptake by transpirational pull over cutting edge: Cuttings (length 4 cm, diameter 0.8 cm) of *Vitis vinifera* cv. “Riesling” were grown for ≈1.5 months in perlite until ≈3–4 leaves were formed. The plants were placed in a vial with 5 mL of a 0.2 wt% nanocarrier dispersion for 7 days at ≈32 °C (Figure 5A). Water was refilled regularly. Each experiment was performed in duplicates or triplicates.
- Nanocarrier injections: *Vitis vinifera* cv. “Riesling” test plants (length: 25–30 cm, trunk diameter: 1.0–1.5 cm, one shoot with leaves: 30–40 cm) were grown in a pot with breeding ground until used (Figure 6B). To investigate the nanocarrier transport, 20 cm above the soil a 1–2 mm wide hole was drilled ≈0.5 cm into the trunk and 0.2 µL of a 10 wt% PFCE‐PLGA dispersion was injected with a 1 mL syringe attached to a metal extension with conus top (Figure 6C). The wound was sealed with grafting wax. The plant was kept for further days at ≈32 °C under regular addition of water until investigated by ^19^F MRI.

##### Methods—Hydrolysis‐Resistance of PLGA Nanocarriers In Planta

The hydrolysis‐resistance of the nanocarriers was tested before the measurement, by mixing 0.1 mL of 1 wt% dispersion with 0.4 mL of “Riesling” wood extract (pH 5.3). After incubation for 7 days, the mixture was analyzed regarding size distribution and morphology by DLS and SEM. As both remained unchanged in comparison to the original nanocarriers, we assume no hydrolysis in the wood extracts.

##### Methods—Inductively Coupled Plasma Emission Spectroscopy

ICP‐OES was performed at an Activa M spectrometer from Horiba to determine the metal content of “wood extracts”, dispersions containing ruthenium‐labeled nanocarriers and to localize the latter after uptake into grapevine cuttings. The samples were prepared as follows:

Before measurement, the wood extracts (0.5 mL) were diluted with (9.5 mL) MililQ water. The content of potassium (spectral line: 766 nm), sodium (spectral line: 590 nm), magnesium (spectral lines: 279 and 384 nm), calcium (spectral lines: 318 nm, 374 nm, 423 nm), sulfur (spectral lines: 182 and 213 nm) and phosphorus (spectral line: 178 nm) was determined subsequently by ICP‐OES.

To determine the metal content of ruthenium‐loaded polystyrene nanocarriers, 0.2 mL of a 1 wt% dispersion diluted with 9.8 mL MilliQ water. The amount of ruthenium was determined using spectral lines at 240, 267, and 273 nm.

To track ruthenium‐loaded nanocarriers after uptake into grapevine cuttings (according to paragraph “Nanocarrier uptake by transpirational pull over cutting edge”, the plants were cut into four 1 cm long segments. Wood and leaves were dried in an oven at 120°C and dissolved in 3 mL of a 1:1 mixture composed of hydrogen peroxide (30%) and concentrated sulfuric acid (“Piranha solution”). After the addition of further 7 mL MilliQ water, the ruthenium content was determined by ICP‐OES (spectral lines: 240, 267, and 273 nm). To quantify the amount of nanocarriers, which were not taken up by the plant, the residual dispersion was analyzed in the same manner.

##### Methods—^19^F MRI Experiments

Image data was recorded on a Bruker BioSpec 117/16 11.7T horizontal bore MRI (Bruker, Germany) with a dual tuned ^1^H/^19^F bird cage coil with a length of 4 cm. For ^19^F imaging, a 3D RARE sequence was used, imaging parameter: TR 1500 ms, TE 6.62 ms, turbo factor 44, 32 averages, matrix 64 × 64 × 16, field of view of 32 × 45 × 32 mm, imaging time 12:48 min. Excitation frequency 470,743 MHz. Co‐registration of ^1^H anatomical images was done with an unspoiled T1‐weighted 2D FLASH, local shimming avoided artifacts in the low water content regions of the stem. Imaging parameters: TE 2.5 ms, TR 268 ms, FA 50deg, 2 averages, matrix 248 × 248 zero‐filling to a 256 × 360 matrix, field of view 32 × 45 mm, 40 1mm thick slices for an imaging time of 2:15 min. To obtain a sufficiently low ^19^F detection threshold to visualize low concentrations of nanocarriers, a high‐field small animal MRI system was used. The coil length did not allow for imaging of the entire stem and shoot simultaneously. Therefore, the length of the shoot was imaged in three separate images, anatomical landmarks, such as notches, allowed for the reconstruction of the entire stem and shoot.

##### Syntheses—Preparation of Ruthenocene‐Loaded Polystyrene Nanocarriers

Polystyrene nanocarriers were produced by free‐radical polymerization in miniemulsion using a modified literature protocol.^[^ ^49^, ^50^ ^]^ For PS‐SDS, hexadecane (8.3 mg, 0.036 mmol), V59 (3.3 mg, 0.017 mmol), and ruthenocene (6.6 mg, 0.029 mmol) were added to freshly distilled styrene (0.2 g 1.920 mmol). After addition of 0.8 mL of an aqueous 3 mg mL^−1^ SDS solution, the mixture was stirred with an IKA Ultraturrax to generate a pre‐emulsion and then sonicated for 2 min (Branson Digital Sonifier W450‐D, 1/4” tip, 70% amplitude) under ice‐cooling. Afterward, the polymerization was allowed to proceed for 24 h at 72 °C. Finally, the polystyrene dispersion was diluted with 5 mL MilliQ water and filtered through a KimWipe to remove macroscopic aggregates.

To exchange SDS against Lutensol AT50, a Lutensol solution (5 mg mL^−1^) was added to the dispersion and the mixture was dialyzed (MWCO 1 kDa) against 500 mL MilliQ water for 3 h (this procedure was repeated five times) to yield PS‐Lut.

To prepare CTMA‐Cl‐stabilized nanocarriers, a CTMA‐Cl solution (5 mg mL^−1^) was added to the previously prepared PS‐Lut nanocarriers and the dispersion was dialyzed (MWCO 25 kDa) against a 500 mL CTMA‐Cl solution (5 mg mL^−1^) for 3 h (this procedure was repeated five times) to obtain PS‐CTMA.

Carboxyl‐functionalized nanocarriers (PS‐COOH) were prepared according to the above mentioned free‐radical miniemulsion polymerization, adding additionally acrylic acid (4 mg, 0.056 mmol) to the dispersed phase prior sonication. For amino‐functionalized nanocarriers (PS‐NH_2_), 2‐aminoethyl methyacrylate hydrochloride (4 mg, 0.024 mmol) was dissolved in water instead. After addition of 0.8 mL of an aqueous 2.5 mg mL^−1^ Lutensol AT50 solution, the mixture was stirred with an IKA Ultraturrax and then sonicated for 2 min (Branson Digital Sonifier W450‐D, 1/4” tip, 70% amplitude) at 0 °C. The polymerization proceeded for 24 h at 72 °C. Afterward, 5 mL MilliQ water was added and the mixture was filtered through a KimWipe. To set the surfactant concentration to 5 mg mL^−1^, the dispersion was dialyzed three times for 3 h against 500 mL of a Lutensol AT50 (5 mg mL^−1^) solution with MWCO of 25 kDa. Finally, all dispersions were set to a concentration of 1 wt% with MilliQ water and characterized by DLS, SEM, ICP‐OES and regarding their *ζ*‐potential.

##### Syntheses—Preparation of Ruthenocene‐Loaded Polystyrene Microcarriers

Polystyrene microcarriers were prepared by free‐radical dispersion polymerization according to Jinhua et al.^[^ ^51^ ^]^ V59 (3.3 mg, 0.017 mmol) and ruthenocene (6.6 mg, 0.028 mmol) were mixed with freshly distilled styrene (0.196 g, 1.885 mmol). To the mixture, an ethanol solution of polyvinylpyrrolidone (40 mg in 1.76 g ethanol, 24 kg mol^−1^) was added. After stirring at 250 rpm for 24 h at 72 °C, the dispersion was centrifuged (30 min, 4000 rpm) and the supernatant was replaced with MilliQ water. For further purification, the beads were additionally washed twice with 5 mL water. The solid content was finally adjusted to 1 wt% by the addition of MilliQ water.

##### Syntheses—Preparation of Kraft Lignin and Lignin Sulfonate Nanocarriers

Lignin nanocarriers were prepared according to our previously reported protocol by crosslinking methacrylated Kraft lignin in a direct miniemulsion.^[^ ^5^ ^]^ Interfacial polyaddition in an inverse miniemulsion was used to prepare hollow lignin nanocarriers from lignin sulfonate sodium salt and toluene diisocyanate following our literature protocol.^[^ ^34^ ^]^

## Conflict of Interest

The authors declare no conflict of interest.

## Supporting information

Supporting Information

## Data Availability

Research data are not shared.
