# Supplementary material for: Targeted Drug Delivery for Sustainable Crop Protection: Transport and Stability of Polymeric Nanocarriers in Plants
Source: Adv Sci (Weinh). 2021 Mar 19;8(11):2100067. doi: 10.1002/advs.202100067 (PMC8188206; doi:10.1002/advs.202100067)
Supplement: Supplementary file 1 — Supporting Information [file ADVS-8-2100067-s001.pdf]

© 2021 Wiley-VCH GmbH

ADVANCED SCIENCE
Open Access

Supporting Information

for *Adv. Sci.*, DOI: 10.1002/advs.202100067

Targeted drug delivery for sustainable crop protection: Transport and stability of polymeric nanocarriers in plants

Sebastian J. Beckers,[a] *Alexander H. J. Staal,*[b] *Christine Rosenauer,*[a] *Mangala Srinivas,* [b,c] *Katharina Landfester,* [a] *Frederik R. Wurm* [a,d]*

Supporting Information for

Targeted drug delivery for sustainable crop protection: Transport and stability of polymeric nanocarriers in plants

Sebastian J. Beckers,[a] Alexander H. J. Staal,[b] Christine Rosenauer,[a] Mangala Srinivas, [b,c] Katharina Landfester, [a] Frederik R. Wurm [a,d]*

[a]*Max-Planck-Institut für Polymerforschung, Ackermannweg 10, 55128 Mainz, Germany.*

[b]*Department of Tumor Immunology, Radboud Institute for Molecular Life Sciences, Radboud University Medical Center, Geert Grooteplein 26/28, 6525GA, Nijmegen, The Netherlands.*

[c] *Cenya Imaging BV, Tweede Kostverlorlenkade 11h, 1052RK, Amsterdam, The Netherlands.*

[d] *Sustainable Polymer Chemistry Group, MESA+ Institute for Nanotechnology, Faculty of Science and Technology, Universiteit Twente, PO Box 217, 7500 AE Enschede, The Netherlands. frederik.wurm@utwente.nl*

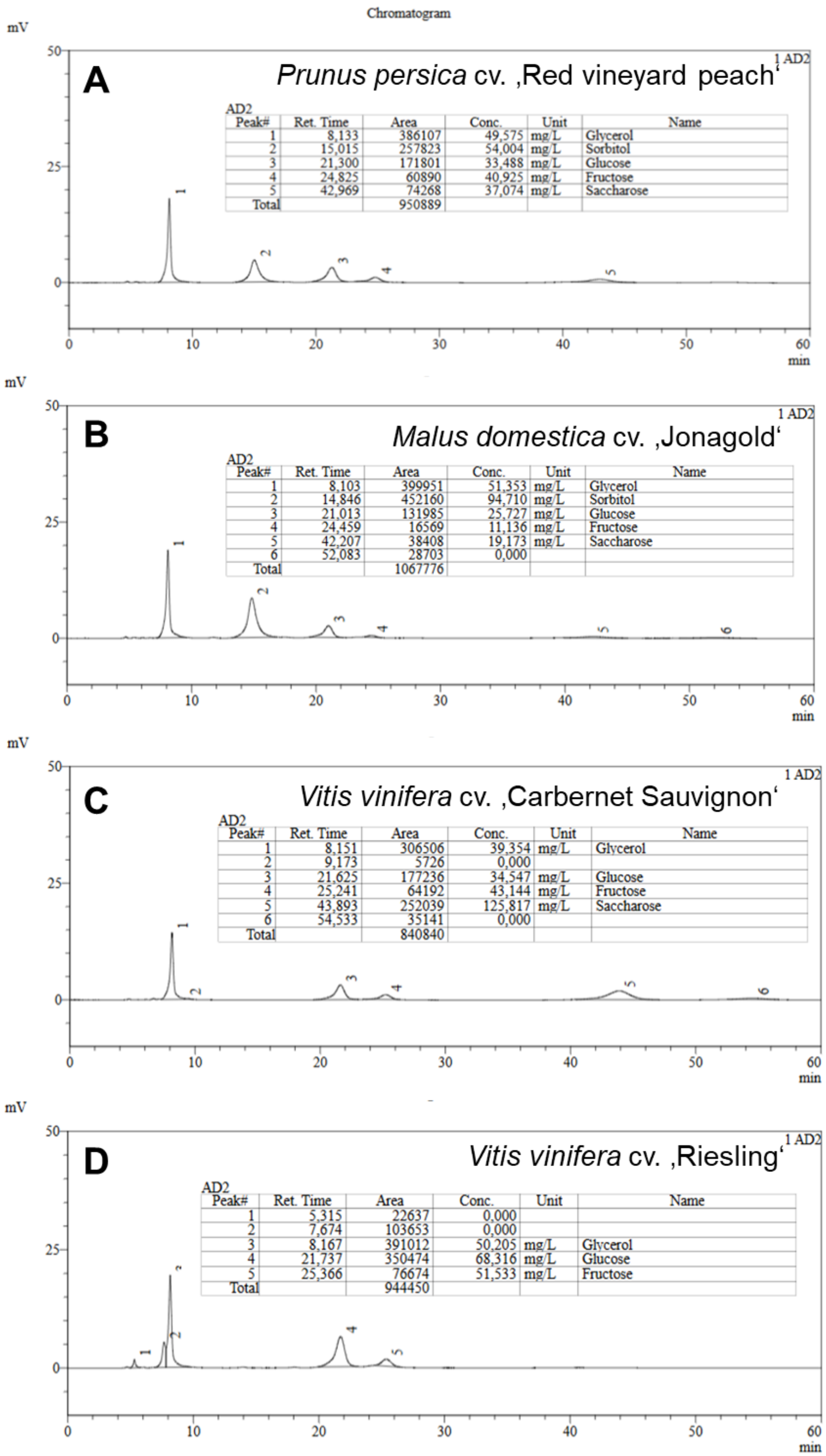

Figure S1: Elugrams measured by HPEAC-PAD of wood extracts from apple, peach and grapevine woodcut.

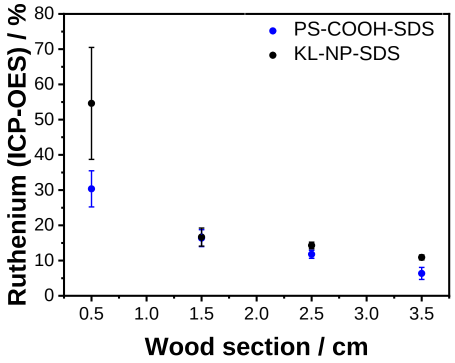

Figure S2: Distribution profile of ruthenium loaded lignin nanocarriers (KL-NP-SDS) in comparison to polystyrene-based nanocarriers (PS-COOH-SDS) after uptake into the trunk of a Riesling cutting. The plants were immersed in a nanocarrier dispersion for 7 days. Ruthenium was quantified by ICP-OES.

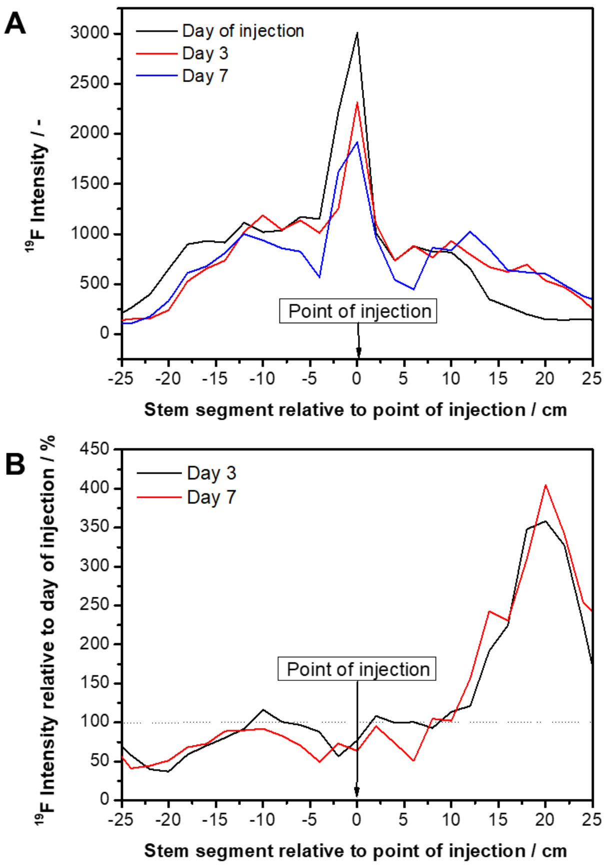

Figure S3: Distribution profile of PFCE-loaded nanocarriers in the trunk of potted Riesling plants after injection with a syringe. A) ^{19}F Intensity relative to point of injection after 0 and 3 days and after a week. B) ^{19}F Intensity related to the intensity measured directly after injection (day 0). A slight movement of upwards the stem was observed.

Table S1: Ruthenium concentrations quantified by ICP-OES in wood segments of 'Riesling' cuttings after uptake of ruthenocene-loaded polystyrene nanoparticles.

Sample ID	Plant segment:	1	2	3	4	Res. Disp.
**PS-SDS-1**	**c(Ru-ICP)**	1.55	0.46	0.26	0.16	0.75
	**Ru%**	48.74	14.47	8.18	5.03	23.58
**PS-SDS-2**	**c(Ru-ICP)**	1.69	0.58	0.24	0.18	0.35
	**Ru%**	55.59	19.08	7.89	5.92	11.51
	**Average**	**52**	**17**	**8**	**5**	**18**
	**Error**	**3.42**	**2.31**	**0.14**	**0.44**	**6.04**
**PS-Lut-1**	**c(Ru-ICP)**	0.98	0.68	0.38	0.15	0.52
	**Ru%**	36.16	25.09	14.02	5.54	19.19
**PS-Lut-2**	**c(Ru-ICP)**	1.00	0.65	0.44	0.31	0.26
	**Ru%**	37.59	24.44	16.54	11.65	9.77
**PS-Lut-3**	**c(Ru-ICP)**	1.20	0.54	0.38	0.14	0.72
	**Ru%**	40.27	18.12	12.75	4.70	24.16
	**Average**	**38**	**23**	**14**	**7**	**18**
	**Error**	**1.70**	**3.14**	**1.57**	**3.10**	**5.97**
**PS-CTMA-1**	**c(Ru-ICP)**	0.05	0.00	0.00	0.00	0.15
	**Ru%**	25.00	0.00	0.00	0.00	75.00
**PS-CTMA-2**	**c(Ru-ICP)**	0.06	0.03	0.01	0.00	0.15
	**Ru%**	24.00	12.00	4.00	0.00	60.00
	**Average**	**25**	**6**	**2**	**0**	**68**
	**Error**	**0.50**	**6.00**	**2.00**	**0.00**	**7.50**
**PS-COOH-1**	**c(Ru-ICP)**	0.56	0.36	0.25	0.10	1.14
	**Ru%**	23.24	14.94	10.37	4.15	47.30
**PS-COOH-2**	**c(Ru-ICP)**	0.75	0.33	0.27	0.15	0.79
	**Ru%**	32.75	14.41	11.79	6.55	34.50
**PS-COOH-3**	**c(Ru-ICP)**	0.80	0.45	0.30	0.19	0.54
	**Ru%**	35.09	19.74	13.16	8.33	23.68
	**Average**	**30**	**16**	**12**	**6**	**35**
	**Error**	**5.13**	**2.40**	**1.14**	**1.71**	**9.65**
**PS-NH2-1**	**c(Ru-ICP)**	0.95	0.30	0.17	0.07	0.42
	**Ru%**	49.74	15.71	8.90	3.66	21.99
**PS-NH2-2**	**c(Ru-ICP)**	0.50	0.11	0.04	0.01	1.63
	**Ru%**	21.83	4.80	1.75	0.44	71.18
**PS-NH2-3**	**c(Ru-ICP)**	0.86	0.38	0.27	0.18	0.47
	**Ru%**	39.81	17.59	12.50	8.33	21.76
	**Average**	**37**	**13**	**8**	**4**	**38**
	**Error**	**11.55**	**5.64**	**4.47**	**3.24**	**23.24**
**PS-Mikro-1**	**c(Ru-ICP)**	0.08	0.04	0.04	0.02	0.02
	**Ru%**	41.03	20.51	20.51	10.26	7.69
**PS-Mikro-2**	**c(Ru-ICP)**	0.17	0.06	0.04	0.02	0.14
	**Ru%**	39.53	13.95	9.30	4.65	32.56
	**Average**	**40**	**17**	**15**	**7**	**20**
	**Error**	**0.75**	**3.28**	**5.61**	**2.80**	**12.43**

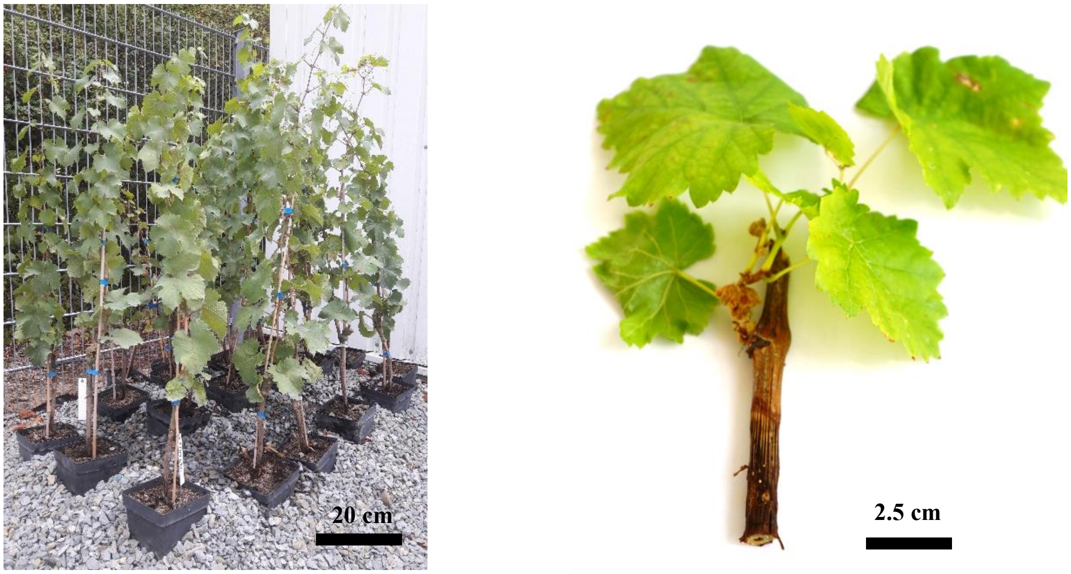

Figure S4: *Vitis vinifera* cv. ‘Riesling’ plants used for *in planta* studies. Into the plants on the left the nanocarriers were injected, while the grapevine cuttings on the right were immersed into a nanocarrier dispersion
